# Prophylactic Tocilizumab Prior to Anti-CD19 CAR-T Cell Therapy for Non-Hodgkin Lymphoma

**DOI:** 10.3389/fimmu.2021.745320

**Published:** 2021-10-12

**Authors:** Paolo F. Caimi, Gabriela Pacheco Sanchez, Ashish Sharma, Folashade Otegbeye, Nausheen Ahmed, Patricio Rojas, Seema Patel, Sarah Kleinsorge Block, Jennifer Schiavone, Kayla Zamborsky, Kirsten Boughan, Antoinette Hillian, Jane Reese-Koc, Mikhail Maschan, Boro Dropulic, Rafick-Pierre Sekaly, Marcos de Lima

**Affiliations:** ^1^ Department of Hematology and Oncology, Cleveland Clinic, University Hospitals Seidman Cancer Center, and Case Western Reserve University, Cleveland, OH, United States; ^2^ Department of Pathology, Emory University, Atlanta, GA, United States; ^3^ Department of Medicine, The University of Kansas, Kansas City, KY, United States; ^4^ Escuela de Medicina, Pontificia Universidad Católica de Chile, Santiago, Chile; ^5^ Dmitryi Rogachev National Medical Research Centre of Pediatric Hematology, Oncology and Immunology, Moscow, Russia; ^6^ Lentigen, A Miltenyi Biotec Company, Gaithersburg, MD, United States

**Keywords:** tocilizumab, CAR- T cells, lymphoma, prophylaxis, cytokine release syndrome (CRS)

## Abstract

Anti-CD19 chimeric antigen receptor T (CAR-T) cells have demonstrated activity against relapsed/refractory lymphomas. Cytokine release syndrome (CRS) and immune effector cell – associated neurotoxicity syndrome (ICANS) are well-known complications. Tocilizumab, a monoclonal antibody targeting the interleukin-6 (IL-6) receptor was administered 1 hour prior to infusion of anti-CD19 CAR-T cells with CD3ζ/4-1BB costimulatory signaling used to treat non-Hodgkin lymphoma patients. Relapsed/refractory lymphoma patients treated with anti-CD19 CAR-T cells were included in this analysis. Cytokine plasma levels were measured by electrochemiluminescence before lymphodepleting chemotherapy, prior to infusion and then on days 2, 4,6, and 14 days after treatment. Twenty patients were treated. Cell products included locally manufactured anti-CD19 CAR-T (n=18) and tisagenlecleucel (n=2). There were no adverse events attributed to tocilizumab. Ten patients had grade 1–2 CRS at a median of 4 (range 3-7) days. There were no cases of grade ≥3 CRS. Five patients had ICANS, grade 1 (n=4) and grade 4 (n=1). Laboratory studies obtained prior to lymphodepleting chemotherapy were comparable between patients with and without CRS, except for interleukin (IL)-15 plasma concentrations. patients with CRS had higher post-infusion ferritin and C reactive protein, with more marked increases in inflammatory cytokines, including IL-6, IL-15, IFN-γ, fractalkine and MCP-1. Fifteen patients (75%) achieved CR and 2 (10%), PR. One-year OS and PFS estimates were 83% and 73%. Prophylactic tocilizumab was associated with low CRS incidence and severity. There were no adverse events associated with tocilizumab, no increase in frequency or severity of ICANS and excellent disease control and overall survival.

## Introduction

pt?>Adoptive immunotherapy with CD19-targeting chimeric antigen receptor (CAR)-T cells is an effective therapeutic strategy against relapsed or refractory B cell malignancies. The chimeric antigen receptor itself is a recombinant T cell receptor composed of an extracellular domain derived from the immunoglobulin variable fragment as single chain (scFv), linked to transmembrane domain and intracellular signaling sequences that are derived from T cells (1). Several second-generation CAR-T cell products, characterized by the combination of the CD3ζ T cell subunit and a costimulatory domain (2) are approved for treatment of B cell malignancies. Axicabtagene ciloleucel (axi-cel), an antiCD19 CAR-T cell product with CD28 costimulatory domain in addition to CD3ζ, currently approved for treatment of relapsed diffuse large B cell lymphoma (DLBCL)and follicular lymphoma (3, 4). Brexucabtagene autoleucel has identical construct to axi – cel but circulating CD19 positive B cells are removed from the starting material prior to manufacture. Brexu-cel is approved for treatment of relapsed mantle cell lymphoma (5). Two products with a different T cell costimulatory domain, 4-1BB are currently approved: tisagenlecleucel (tisa-cel), approved for treatment of relapsed/refractory pediatric B cell acute lymphoblastic leukemia and relapsed/refractory diffuse large B cell lymphoma (6, 7); and lisocabtagene maraleucel (liso-cel), which also has 4-1BB costimulatory domain but differs from tisa-cel in that it is formulated with equal target doses of CD4+ and CD8+ CAR-T cells. Liso-cel is approved for relapsed/refractory diffuse large B cell lymphoma. Several CAR-T cell products have received FDA approval for treatment of relapsed diffuse large B cell lymphoma (DLBCL) (8) (6) (3, 8) or mantle cell lymphoma (9).

Treatment with CAR-T cells is associated with well described acute adverse events, including cytokine release syndrome (CRS) and neurotoxicity, termed immune effector cell associated neurologic syndrome (ICANS). CRS and ICANS occurred in 58% and 21% respectively of lymphoma patients treated with tisa-cel (6), 93% and 64%, respectively after axi-cel (3), and 42% and 30% after liso-cel (8). Higher grades of immune adverse events may be life threatening, require clinical support that may include critical care interventions and thus limit the applicability of these immunotherapies (10).

Acute complications of CAR-T cell therapy are the result of rapid CAR-T cell expansion (11) and a hyperinflammatory state related to cell activation, in conjunction with other compartments of the immune system, such as monocytes and macrophages (12, 13). Interleukin (IL)-6 is a central mediator of cytokine responses in CRS and ICANS (14), with other cytokines and chemokines involved in propagation of the inflammatory signal. IL-6 interacts with its receptor (IL-6R) in either membrane-bound form, leading to “classic” IL-6 signaling after interacting with gp130, or soluble in plasma, where the IL-6–IL6R complex interacts with gp130 expressing cells in “trans” IL-6 signaling (15, 16).

Tocilizumab is a humanized monoclonal antibody that binds the IL-6R in both its soluble and membrane – bound forms, preventing both “trans” and “classic” IL-6 signaling (17). Earlier CAR-T trials in acute lymphoblastic leukemia identified that CRS could be successfully treated with tocilizumab (18). Subsequent studies in relapsed non–Hodgkin lymphoma (NHL) showed that the use of tocilizumab for treatment of CRS did not affect disease control (3, 6) or CAR-T cell expansion (19).

In May of 2018, we modified our institutional CAR-T premedication procedures to include tocilizumab administration prior to cell administration. Here we report outcomes of NHL patients receiving prophylactic tocilizumab and anti-CD19 CAR-T cells with CD3ζ/4-1BB costimulatory signals.

## Methods

Patients were treated with CAR-T cells at our institution. Data was collected under the University Hospitals Seidman Cancer Center Hematologic Malignancies and Stem Cell Transplant Database Protocol (DATA 0213). All subjects received antiCD19 CAR-T cells with CD3ζ and 4-1BB costimulatory domain.

CAR-T cells were either from commercial sources (tisagenlecleucel, Kymriah^®^) delivered as standard of care, or locally manufactured antiCD19 CAR-T cells delivered within a phase I clinical trial (NCT03434769, IND 17932). In this trial CAR-T cells were manufactured on the automated CliniMACS Prodigy^®^ device, using a lentiviral vector [LTG 1563, Lentigen a Miltenyi Biotec company (Gaithersburg, MD)]. The lentiviral vector contained a second generation anti-CD19 CAR, with a scFv FMC63 CD19 targeting domain, a CD8 hinge region, TNFRSF19-derived transmembrane region, 4-1BB costimulatory domain and CD3ζ chain intracellular signaling domain. The main difference of this construct with other CD3ζ/4-1BB constructs lies on the transmembrane domain. We have previously reported on the successful point of care manufacture and safety of anti-CD19 CAR-T cells using this construct (20).

The institutional procedure prior to infusion of all immune effector cells (commercial or clinical trial) included: acetaminophen 650mg orally, diphenhydramine 50mg intravenously (IV) or orally, and tocilizumab, 8mg/kg IV, infused over 1 hour. Prophylaxis procedures were completed 1 hour prior to infusion of anti-CD19 CAR-T cells. Patients treated with site manufactured antiCD19 CAR-T cells received lymphodepletion with fludarabine 25 mg/m^2^ daily for three days (days -5, -4 and -3), and cyclophosphamide 60 mg/Kg on day -6. lymphodepletion for patients treated with tisagenlecleucel consisted of fludarabine (25 mg/m^2^) and cyclophosphamide (250mg/m^2^) intravenous daily for three days (days -5, -4 and -3).

Cytokine release syndrome was graded according to the Lee scale (21, 22). Immune effector cell associated neurotoxicity syndrome was graded using NCI Common Terminology Criteria for Adverse Events, version.5.0 (https://ctep.cancer.gov/protocoldevelopment/electronic_applications/ctc.htm)

Patients were monitored daily, 6 days a week from day 1 through day 30 after infusion, including physical examination, laboratory studies, writing evaluation, and neurologic evaluation using the neurological assessment score (CARTOX-10) (22). If patients developed CRS or ICANS, they were treated according to our standard of care, which included monitoring and tocilizumab treatment for CRS Grade 2 or higher (according to the Lee scale). Patients with ICANS ≥ Grade 2 (with or without concomitant CRS) were administered systemic corticosteroids (10, 21). Monitoring protocols and institutional guidelines for treatment of CRS and ICANS were not modified after the introduction of tocilizumab prophylaxis except for limiting additional tocilizumab dosing for CRS treatment to one dose of tocilizumab (i.e. maximum total of 2 doses of tocilizumab). Our institutional guidelines were modified for CRS persisting after one dose of tocilizumab to be managed with other agents, including siltuximab, anakinra and corticosteroids. Research was conducted in accordance with the Helsinki declaration, patient data and samples were obtained under IRB approved protocols DATA 0213, CASE12Z05, CASE 2417.

Cytokines were measured prior to cell infusion and then on days 2, 4,6, and 14 days after CAR-T cells. Measurements were done with electrochemiluminescence (MesoScale Diagnostics, Rockvile, MD). Tumor burden and disease response were assessed with positron emission tomography scans on day 30, 60 and 90. Disease response was assessed according to the Lugano Response Criteria (23).

In patients treated with locally manufactured CAR-T cells, CAR-T cell expansion was measured with quantitative PCR (qPCR). Briefly, for each patient sample collected, peripheral blood mononuclear cells (MNCs) were isolated using Ficoll centrifugation. DNA was isolated from MNCs using the Qiagen DNeasy kit (Qiagen, Germantown, MD) and DNA concentration determined by PicoGreen assay (ThermoFisher, Waltham, MA). Cells containing the provirus were detected using 40 cycles of qPCR targeting the lentiviral backbone Rev response element (RRE) using TaqMan Universal PCR Master Mix (Life Technologies) and primers and FAM- conjugated probe from Lentigen Corporation (Gaithersburg, MD). Vector copies are determined by comparison to standard curve of pLKO.1 puro plasmid. RRE is converted to copies per cell using the calculation: copy number/ng DNA x 6 pg DNA per cell ÷ 1000 pg/ng.

Comparisons were done using Fisher test for categorical variables and Wilcoxon test for continuous variables. Overall and progression-free survival (PFS) estimates were done using the Kaplan Meier method (24). All statistical analyses were performed using R and its packages (http://cran.r-project.org). Changes in cytokine concentration were calculated with the pre-lymphodepletion baseline as reference value. Heatmaps of cytokine concentrations change from baseline were illustrated using the ggplot R package. Area under the curve (AUC) calculations of CAR-T expansion and cytokine concentration over time were done using the DescTools R package and compared using Wilcoxon test, as described by Kadauke and colleagues (25). Survival comparisons between groups were done with log - rank test using the package survminer.

## Results

### Baseline Patient, Disease and Infused Product Characteristics

Twenty patients received prophylactic tocilizumab prior to antiCD19 CAR-T cell infusion (locally manufactured CAR-T, n = 18; tisagenlecleucel, n = 2) for treatment of relapsed refractory NHL (**Table 1**).

**Table 1:** Patient Characteristics.

Characteristic	Overall N (%)
Age at CAR-T infusion, years, median (range)	56 (33 – 76)
Gender	
Male	15 (75)
Female	5 (25)
Diagnosis	
Diffuse large B cell lymphoma	8
Transformed follicular lymphoma	1
Follicular lymphoma	4
Mantle cell lymphoma	6
Burkitt lymphoma	1
Previous treatment regimens, median (range)	4 (2-7)
Bulky disease	
7.5 - 10cm	3
> 10cm	1
Primary refractory disease	8
Disease refractory to last line of therapy prior to CAR-T	15

Most patients were male (n=15, 75%) and the majority had diffuse large B cell lymphoma (DLBCL) or transformed indolent lymphoma (n=9, 45%), while six patients (30%) had mantle cell lymphoma and four (20%) had follicular lymphoma. Median number of previous treatment regimens was 4 (range, 2-7) and 10 (50%) had a previous autologous stem cell transplant. Eight (40%) patients had primary refractory disease and 18 (90%) had disease that was refractory to the last line of therapy. The median total metabolic tumor volume was 61 ml (range 2 - 1502). Six patients (30%) had elevated lactate dehydrogenase prior to lymphocyte collection.

Seventeen patients received fresh product infusion, whereas three patients (tisa-cel, n=2, locally manufactured CAR-T, n=1) received cryopreserved product. The median time from lymphocyte collection to infusion was 13 days (range 9-42). CAR-T cell dose for patients treated with locally manufactured product was 0.5 x 10^6^ cells/kg in one patient, 1 x 10^6^ cells/kg in 11 patients and 2 x 10^6^ CAR-T cells/kg in 6 patients. Specific cell dose data for patients treated with tisagenlecleucel was not available, but one patient was treated under an expanded access protocol due to lower cell product viability observed during post manufacture testing.

Among patients treated with locally manufactured CAR-T cells, there were no differences in the incidence of CRS for the different CAR-T cell doses (**Table 2**). There were no differences in the median culture expansion or in the percentages of CD4 and CD8 positive CAR-T cell in the infused products of patients who developed CRS *vs*. those who did not (**Table 2**).

**Table 2:** Cell product characteristics among patients with locally manufactured CAR-T cells (n = 18).

	CRS (n = 8)	No CRS (n = 10)	p value
CAR-T cell dose			
0.5 x 10^6^/kg	1	0	0.14
1.0 x 10^6^/kg	3	8	
2.0 X 10^6^/kg	4	2	
Product composition (%), median, range			
CD4+	66 (40-84)	66 (52-88)	0.8
CD8+ CD4+, median (range)	34 (16-60)	34 (12-48)	
Culture expansion, fold, median (range)	30 (3 – 47)	25 (14 – 41)	0.9

### Safety

CRS was observed in 10 (50%) patients, of which it was grade 1 in 7 patients (35%) and grade 2 in three patients (15%). There were no grade ≥ 3 CRS events. The median time from CAR-T cell infusion to CRS was 4 days (range, 3-7) (**Table 3**).

**Table 3:** Summary of immune effector cell toxicities.

	Number of subjects (%)
Cytokine release syndrome	
Grade 1	7 (35%)
Grade 2	3 (15%)
Time from infusion to CRS onset, days, median (range)	4 (3-7)
Neurotoxicity	
Grade 1 – 2	4 (20%)
Grade 3 – 4	1 (5%)
Time to neurotoxicity onset, days, median (range)	7 (5-9)
Intensive Care Unit transfer	1 (5%)
Mechanical ventilation	0
Post infusion tocilizumab, n (%)	5 (25%)
Post infusion corticosteroid, n (%)	4 (20%)
Post infusion anakinra, n (%)	2 (10%)
CRS: cytokine release syndrome	

ICANS occurred in five patients (25%). ICANS was of grade 1-2 in four patients (20%), including headache in one patient and decreased CARTOX scores in three patients [dysgraphia (n=1), mild disorientation (n = 2)]. One patient (5%) developed grade 4 ICANS characterized by encephalopathy and aphasia that lasted 36 hours, with complete response to corticosteroids. Median time from CAR-T cell infusion to ICANS was 7 days (range, 5-9).

There were no adverse events attributable to tocilizumab.

CRS and ICANS treatment included tocilizumab (n=5; including 2 subjects with grade 1 CRS and 3 with grade 2 CRS). Corticosteroids were used to treat ICANS (n=3) and grade 2 CRS in an older patient (n=1). Anakinra was used in two subjects who had ICANS (grade 1 and grade 4). Intensive care monitoring was required in the patient with grade 4 ICANS due to global aphasia and agitation.

CAR T-related hematologic side effects were common, with grade ≥ 3 hematologic toxicity observed in all patients, including grade ≥ 3 neutropenia, anemia and thrombocytopenia in 20 (100%), 11 (55%) and 12 (60%) patients, respectively. Five (25%) patients had grade ≥ 3 hematologic toxicities that persisted beyond day 30 but only one subject had not experienced resolution at the time of data cutoff, 90 days after CAR-T cell infusion.

### Laboratory Parameters, CAR-T Cell Expansion, and Plasma Cytokine Concentrations

**Figure 1:**
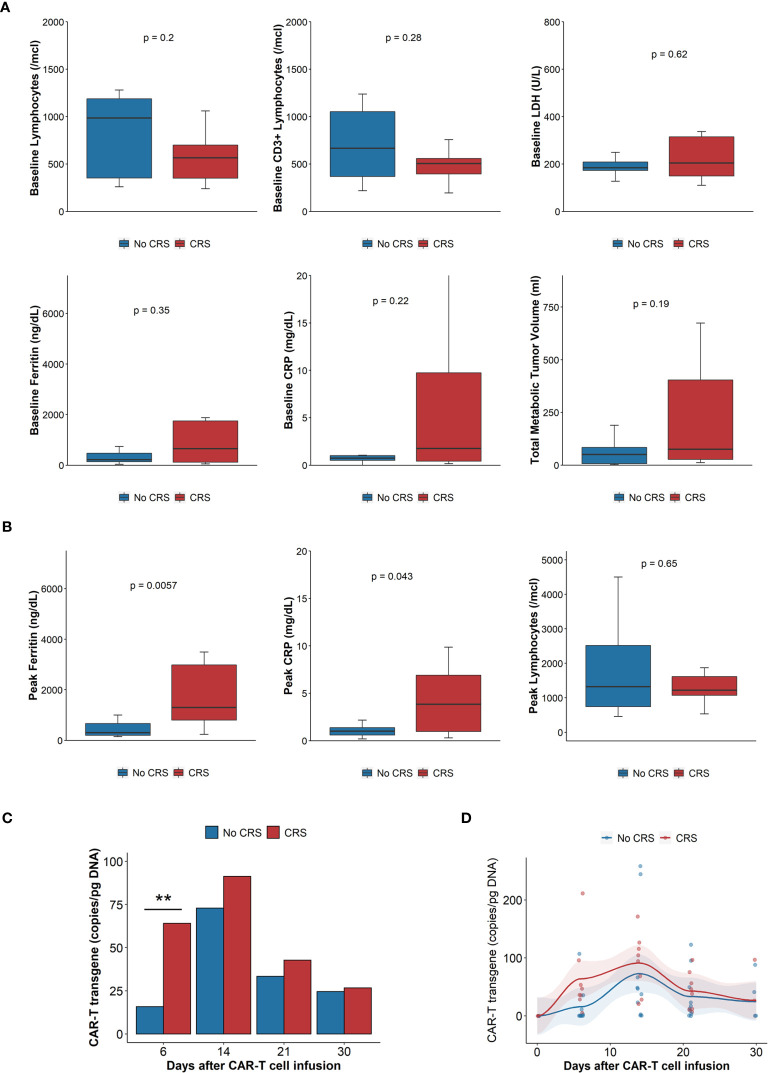
Laboratory parameters, tumor burden and CAR-T cell expansion. **(A)** Baseline laboratory parameters (n = 20): clockwise: absolute lymphocyte count, CD3+ lymphocytes, LDH, total metabolic tumor volume, CRP, ferritin. Comparisons done with Wilcoxon rank sum test. **(B)** Peak laboratory parameters: Ferritin, CRP, absolute lymphocyte count. Blue boxes, bars, dots and lines represent results of patients without CRS, red boxes, bars, dots and lines those with CRS. CRP: C reactive protein; CRS: cytokine release syndrome; LDH: Lactate dehydrogenase. Comparisons done with Wilcoxon rank sum test. **(C)** Mean CAR-T transgene expansion, measured by qPCR, 6, 14, 21 and 30 days after infusion. N = 13. Comparisons done with Wilcoxon rank sum test. **p < 0.01. **(D)** Scatterplot of CAR-T expansion measured by qPCR, trends generated by locally estimated scatterplot smoothing (loess), AUC of CAR-T transgene (copy/pg DNA x days), n = 13, compared with Wilcoxon rank sum test, p = 0.16. Blue boxes, bars, dots and lines represent results of patients without CRS, red boxes, bars, dots and lines those with CRS. AUC, Area under the curve; CRP, C reactive protein; CRS, cytokine release syndrome; LDH, Lactate dehydrogenase qPCR, quantitative polymerase chain reaction.

Baseline total and T cell lymphocyte counts and plasma concentrations of lactate dehydrogenase (LDH), ferritin or C – reactive protein (CRP) were not statistically different in patients experiencing CRS compared with those without CRS (**Figure 1A**). There was a trend towards higher tumor metabolic volume (TMTV) in patients experiencing CRS, however, as this was driven by a few patients with high TMTV, the difference was not statistically significant. Median peak ferritin and CRP levels were higher for patients experiencing CRS, 1866 *vs*. 301 ng/dL (p<0.01) and 3.8 *vs*. 1.0 mg/dL (p=0.04), respectively (**Figure 1B**). There were no differences in the peak lymphocyte counts (1,364 *vs*. 1,888/mcl, p=0.2) after CAR-T cell infusion (**Figure 1B**). Patients who developed CRS had more rapid CAR-T expansion, with higher number of CAR-T transgene detected in peripheral blood on day 6 (**Figure 1C**), but overall expansion over the first month was not different (**Figure 1D**).

**Figure 2:**
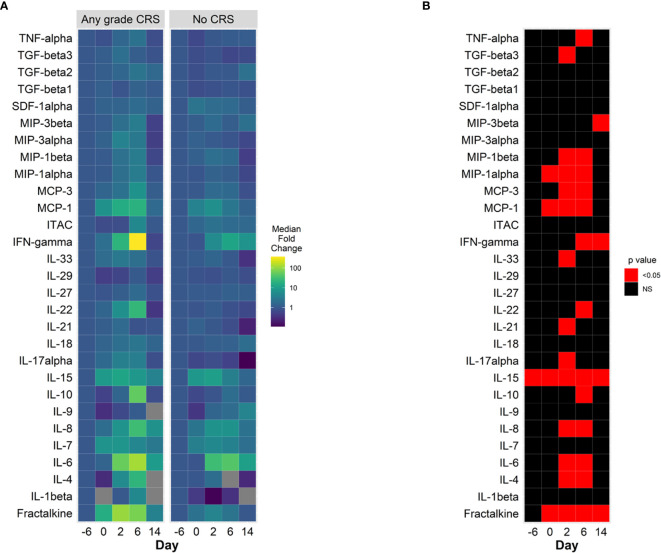
Cytokine changes in CAR-T cell patients treated with prophylactic tocilizumab, (n = 13). **(A)** Median fold change from baseline (pre-lymphodepletion) over time in plasma cytokine concentrations in patients with CRS (left panel) and without CRS (right panel). Days are represented on the horizontal axis. Larger increases from baseline are shown in the green – yellow spectrum. Gray tiles represent missing values. **(B)** Comparisons of mean plasma cytokine concentrations of patients with CRS and without CRS (Wilcoxon test). Red tiles denote p values < 0.05. Not significant values (NS) are noted with black tiles.

Comparison of baseline (prior to lymphodepleting chemotherapy) cytokine concentrations showed that interleukin (IL) 15 was the only cytokine with statistically significant different concentration in patients who developed CRS (**Figures 2A, B**, **Supplemental Figure 1**). On day 0, prior to CAR-T cell infusion, several cytokines had increased plasma concentrations, including IL-15, monocyte chemoattractant protein 1 (MCP-1, CCL7), macrophage inflammatory protein (MIP) 1α and fractalkine, with more marked and statistically significant increases in patients who subsequently developed CRS (**Figure 2B**). On days 2 and 6 after CAR-T infusion, patients had further increases in cytokine plasma concentration, with a pattern denoting immune activation and pro-inflammatory state, including further increases in IL-6 and interferon gamma (IFNγ) with further increases in chemokines MCP-1 and other monocyte – derived chemokines, including MIP1α and MIP1β. Patients who developed CRS had statistically significant higher plasma concentrations of these proteins, in many cases prior to developing symptoms (**Figure 2B**, **Supplemental Figure 1**). Other cytokine levels were not different between patients with and without CRS (**Supplemental Figure 2**). A similar pattern of increases in inflammatory cytokines, with more marked increases in IFNγ and IL-6 can be observed in patients presenting CRS grade 2, compared to patients with grade ≤ 1 (**Supplemental Figure 3**). Patients with ICANS of any grade appeared to have a different pattern of cytokine increases, with less marked elevations of IL-6 and IFNγ compared with those without ICANS, although with higher baseline concentrations of IL-15, tumor necrosis alpha (TNFα) and lower chemokine CXCL12 (SDF1a**) (** **Supplemental Figure 4**).

**Figure 3:**
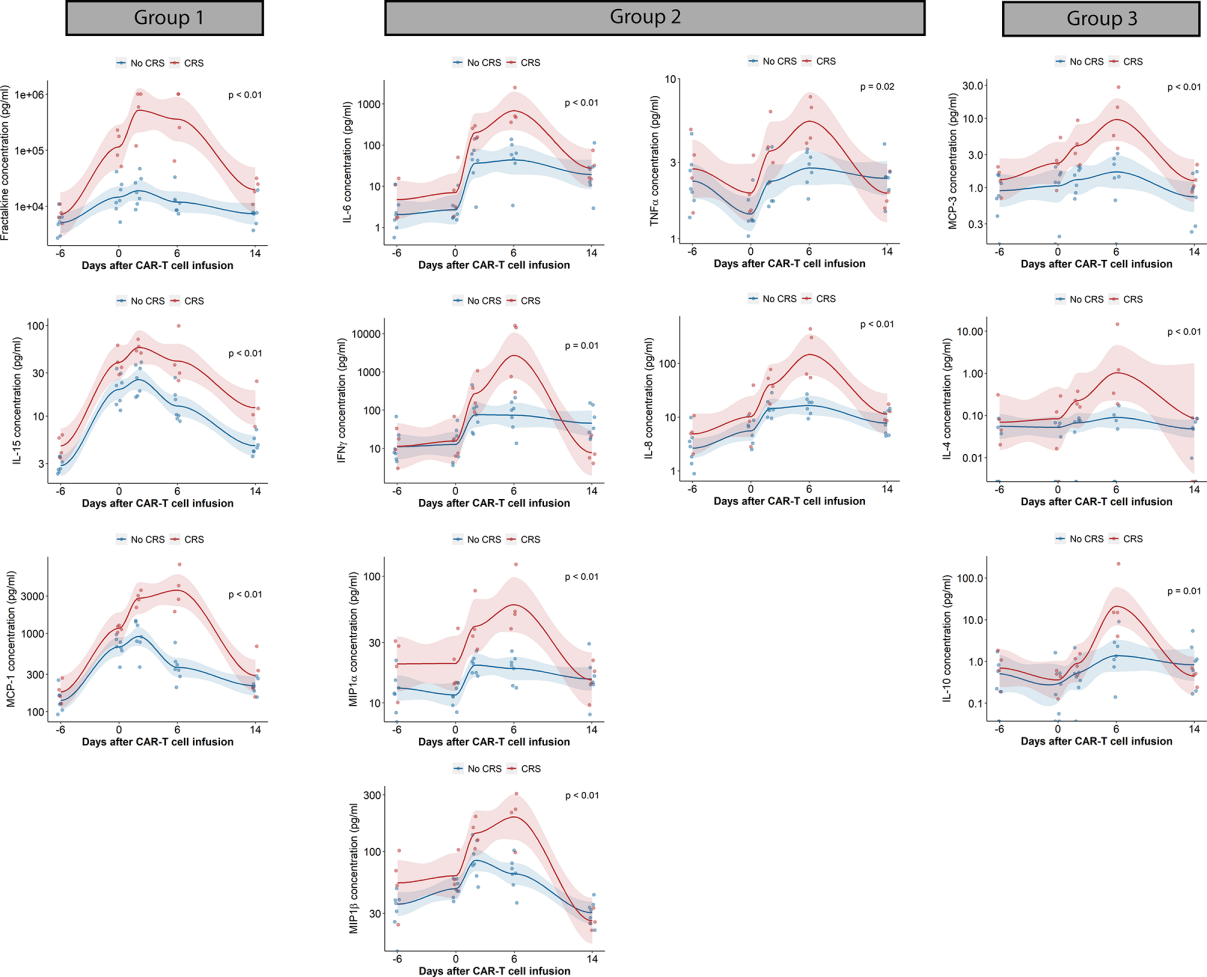
Cytokine changes in CAR-T cell patients treated with prophylactic tocilizumab (n = 13). Scatterplots of cytokine concentrations measured by electrochemiluminescence. Trends generated by locally estimated scatterplot smoothing (LOESS). AUC of cytokine concentration (pg/mL x days) compared with Wilcoxon rank sum test. Blue dots and lines represent patients without CRS, red dots and lines represents patients with CRS. AUC, Area under the curve; CRS, cytokine release syndrome; INFγ, interferon gamma; IL, interleukin; MCP, monocyte chemoattractant protein; MIP, Macrophage inflammatory protein; TNFα, tumor necrosis factor alpha. A. Boxplot of time – based changes in cytokine concentrations with statistically significant differences between patients with CRS (red) and without CRS (blue). Comparisons done with Wilcoxon test.

The AUC of cytokine concentrations over the first 14 days was also evaluated. Cytokines with statistically significant elevated AUC in patients with CRS included fractalkine, IFNγ, IL-4, IL-6, IL-8, IL-10, IL-15, MCP-1, MCP-3, MIP1α, MIP1β and TNFα. Cytokines could be grouped according to their pattern of increase from baseline until day 14 after CAR-T cell infusion (**Figure 3**): the first cytokine group was characterized by an increase in plasma concentration after conditioning chemotherapy followed by sustained elevation through the first week after CAR-T cell therapy (group 1: fractalkine, IL-15, MCP-1); a second group of cytokines was characterized by absence of increase with conditioning, followed by a rapid increase in plasma concentration after CAR-T cell infusion (group 2: IL-6, IFNγ, MIP1α, MIP1β). A third cytokine group can be observed presenting neither changes in plasma concentration during conditioning nor abrupt increases in plasma concentration after CAR-T cell infusion (group 3: MCP-3, IL-4, IL-10). TGFβ1 was the only cytokine with a statistically significant increase in plasma concentration AUC in patients with ICANS (data not shown). We did not find statistically significant differences in the AUC of plasma cytokine concentrations according to disease response.

## Discussion

In this report we show that prophylactic tocilizumab prior to anti-CD19 CAR-T cells with CD3ζ/4-1BB costimulatory signaling is feasible and is associated with a low incidence of high–grade CRS without an increase in ICANS rates or deleterious impact on cell product efficacy. The incidence of CRS was 50% and all instances were of grade 1 or 2. The pivotal JULIET trial of tisagenlecleucel reported an incidence of all – grade and grade ≥3 CRS of 58% and 22%, respectively (6), but this trial conducted CRS grading using the University of Pennsylvania CRS scale. Schuster and colleagues conducted retrospective re-grading of the CRS events of patients participating in the JULIET trial with 17.1% of patients experiencing grade ≥ 3 CRS graded by the Lee criteria (26). Pennisi and colleagues conducted a retrospective study comparing multiple CRS grading scores and observed that University of Pennsylvania scale tends to assign a larger proportion of patients to grade 3 CRS than the ASTCT or Lee criteria, primarily because of fluid – responsive hypotension and/or use of low dose oxygen for hypoxemia or dyspnea (27). In their analysis, when using the Lee or ASTCT grading systems, patients treated with tisagenlecleucel did not experience grade 3 CRS (27). Pasquini and colleagues reported on the results of a prospective CIBMTR registry including 155 non Hodgkin lymphoma patients treated with tisagenlecleucel (28), graded with the ASTCT criteria, which have high concordance with the Lee criteria used in our study (27). All grade and grade ≥ 3 CRS was observed in 45% and 4.5% of patients, respectively (28). A recent report from the Spanish GETH/GELTAMO groups of NHL patients treated with tisagenlecleucel with CRS graded according to the ASTCT criteria showed an overall incidence of CRS of 71% and 5% incidence of grade ≥ 3 CRS (29). The decreased incidence of severe CRS in these later studies can be considered to be secondary to more frequent and earlier use of tocilizumab and corticosteroids and increased experience with CAR-T cell therapies and toxicity management (30). Despite the differences in CRS grading, the absence of high-grade CRS, the low requirement of intensive care management and absence of endotracheal intubation or mechanical ventilation suggest that these severe events can be prevented with early, pre-infusion prophylactic measures.

In a group of eight patients who also received anti-CD19 CAR-T cells with CD3ζ/4-1BB co-stimulatory signaling at our center before implementing prophylactic tocilizumab we observed all-grade CRS in 6/8 (75%) while grade 2 or higher occurred in five (62.5%), including one patient who experienced fatal CRS. Among 7 response evaluable patients, 5 (71.4%) had response, including 4 (57.1%) with complete response. Patients treated with prophylaxis had statistically significantly lower peak post infusion ferritin and C reactive protein, with comparable peak lymphocyte counts. Of note, IL-6 plasma concentrations on day 2 were higher among patients treated with prophylactic tocilizumab (31). While these comparisons are suggestive of the benefit of prophylactic tocilizumab in this patient population, they must be interpreted with caution, as the cohorts were not simultaneous and non – randomized, and patients treated without prophylaxis received CAR-Ts at an earlier time – point, when experience with the toxicity of this therapies was limited at our center.

The role of tocilizumab in the treatment of CRS has been well established (19), highlighting the central role of IL-6 signaling in its pathophysiology. Animal studies have replicated CAR-T cell-associated CRS and neurotoxicity (13), indicating the relevance of bystander monocytes as sources of key cytokines, including IL-6 and IL-1. Clinicopathologic studies conducted on tissues from a patient that died after CRS also suggest that endothelial cells express IL-6 (32). Norelli and colleagues observed that tocilizumab administration to mice prevented CAR-T associated CRS but not neurotoxicity (13).

Early clinical observations in the treatment of CRS indicated that tocilizumab could control fever and hemodynamic instability, only to be followed by ICANS (21). The mechanisms postulated for the ensuing ICANS included: direct neurologic effect of IL-6, observed in neurodegenerative or systemic inflammatory disorders; elevated IL-6 levels in the CNS; and decreased clearance of IL-6 after tocilizumab blockade of IL-6R, leading to transient increases in IL-6 (33, 34). Subsequent studies have shown that multiple cytokines in addition to IL-6 are significantly elevated in the serum and CSF of patients presenting ICANS (35). While the mechanism of ICANS is not completely understood, it appears to be mediated by cytokines, in the context of endothelial cell dysfunction and blood brain barrier integrity disruption (35, 36). Tocilizumab is not effective in treatment of CAR-T neurotoxicity in mouse models (13), in part because it mediates, through IL-6R displacement and decreased IL-6 clearance, an increase in IL-6 serum concentration and also because it does not cross the blood brain barrier (37). However, antecedent severe CRS is the most significant risk factor for development of ICANS (37), possibly because it is associated with marked increases in serum concentration of inflammatory cytokines. It can be postulated that prevention of severe CRS through prophylactic measures, could lead to lower rates of ICANS.

Subsequent clinical trials of axi-cel (3) and tisa-cel (6) confirmed the profile of CAR-T cell mediated CRS, without a reported association between tocilizumab use and increased incidence of ICANS. A safety expansion cohort of NHL patients treated in the ZUMA-1 trial was treated with prophylactic levetiracetam on day 0 (750mg twice daily) and tocilizumab two days after axi-cel infusion (38). The incidence of severe CRS (graded with NCI CTCAE 4.03) was lower than observed without prophylaxis, whereas ICANS incidence was not decreased, with a potential increase in grade ≥ 3 events. This decrease in CRS severity observed with day 2 tocilizumab infusion after axi-cel is compatible with our observations, whereas the persistence in ICANS may be secondary to differences in the toxicity profile of CD3ζ-CD28 *vs*. CD3ζ-41BB CAR-T products. In addition, the later timing of the prophylaxis strategy may also have affected the incidence of adverse events, as cytokine changes can be observed as early as day 2. Earlier IL-6 axis blockade, as done in our patient cohort may prevent further development of the cytokine storm, impeding reaching a threshold that leads to a self-sustaining inflammatory state.

Investigators from the Children’s Hospital of Philadelphia reported the results of a prospective clinical trial of risk-adapted, pre-emptive tocilizumab therapy after tisa-cel in children and young adults with acute lymphoblastic (ALL) (25). Patient with high tumor burden (defined as 40% or more blasts in bone marrow aspirate)(n = 15), were administered tocilizumab (8-12mg/kg) at the time of persistent fever, defined as two temperature measurements ≥ 38.5°C in a 24 hour period. Treatment of CRS (graded with the University of Pennsylvania scale) for this cohort and for the low tumor burden group was done per institutional standards. All 15 high tumor burden patients received pre-emptive tocilizumab and 4/15 (27%) developed grade 4 CRS at a median time to CRS of 2 days (range 2/7). Two of 55 (3.6%) low tumor burden patients developed grade 4 CRS. Immune effector cell – associated neurotoxicity syndrome was observed in 9/15 patients in the high tumor burden (60%) and 10/55 (18%) in the low tumor burden, with grade 3-4 events observed in 4 patients (2 in each cohort). Comparison with a historical cohort of high tumor burden patients showed pre-emptive tocilizumab resulted in lower rates of grade 4 CRS and lower number of additional doses of tocilizumab, with comparable rates of corticosteroid use. There were no differences in the rates of grade ≥ 2 neurologic events (53% *vs*. 54%) and efficacy was also similar between the two cohorts. Molostova and colleagues reported on the results of a phase I trial of point of care manufacture of antiCD19 CAR-T cells for pediatric ALL. Thirty-seven patients were treated with CD19 targeted CAR-T cells received prophylactic tocilizumab (8mg/kg) prior to CAR-t cell infusion. CRS, graded with the Lee scale, was observed in 23 patients but only two patients had grade ≥ 3 CRS. Fifteen patients (40%) had neurologic toxicity, grade ≥ 3 in 5 patients, 2 of whom died (39). The results of these two studies in ALL patients treated with CAR-T cell therapy suggest that both pre-infusion prophylaxis and fever – reactive, pre – emptive therapy may result in higher rates of prevention of severe CRS in ALL patients, without any clear positive or negative effect on the incidence of ICANS. It is possible that the higher rates of severe CRS in patients managed with pre-emptive *vs*. prophylactic strategies are the reflection of a cytokine storm that starts early after CAR-T cell infusion and escalates prior to the development of fever.

Although the risk of CRS and ICANS after CAR-T cell therapy differ in children with ALL and adults with NHL, these results align with our observation that early use of tocilizumab decreases high-grade CRS rates without worsening the frequency or severity of ICANS (25). The tumor-burden risk stratification presented by the investigators from the Children’s Hospital of Philadelphia aims towards a risk - adapted approach that can moderate the potential toxicities and costs associated with indiscriminate prophylaxis. While tumor burden is associated with risk of severe CRS in NHL, several groups are investigating the use of baseline, pre – lymphodepletion laboratory parameters as risk predictors of CRS and ICANS. More recently, investigators have evaluated the use of the Endothelial Activation and Stress Index (EASIX) [lactate dehydrogenase (U/L(x creatinine (mg/dL)/platelet count (10^9^ cells/L)] (40), with either use of CRP concentration instead of creatinine (modified EASIX, mEASIX) (41) or in combination with ferritin and CRP concentrations (EASIX-FC) (42). These modifications of the EASIX score can predict the risk of CRS and) ICANS and mEASIX can also predict disease response (41). Pre-lymphodepletion risk stratification may permit patient selection for the prescription of pre-infusion prophylactic strategies, including tocilizumab, for patients identified to have high risk of CRS.

We observed that patients who developed CRS had higher concentrations of IL-15 prior to lymphodepletion and at all time points after conditioning. Post-conditioning increases in IL-15 plasma concentration have been identified as an important factor affecting CAR-T cell expansion, anti-tumor activity and toxicities including ICANS (43) and CRS (44). The relevance of IL-15 in anti-tumor immune response, CAR-T cell activation and differentiation into persisting T cell subsets is increasingly recognized (45, 46). Our finding of higher pre-conditioning plasma concentrations of IL-15 in patients experiencing CRS could have clinical relevance and highlights the influence of patient – specific factors in the development of toxicities as well as a specific cytokine response. Moreover, as newer predictive models of CRS are developed, measurement of baseline cytokine concentrations, including IL-15, may represent a valuable addition to indices such as the EASIX. Additional research will be needed to validate the role of baseline IL-15 levels and to identify applicable cutoff levels.

We identified the existence of several patterns of cytokine changes through the conditioning and first 14 days after CAR-T cell infusion. These patterns suggest the existence of a specific sequence in the inflammatory cytokine responses to conditioning chemotherapy and then CAR-T cell infusion and highlight the additional relevance of the timing of specific inhibitory interventions.

This study is limited by the heterogeneity of the products used, and the absence of a matched control group not receiving prophylactic tocilizumab. In addition, given the inclusion of patients with an investigational, locally manufactured product that was infused fresh, there are no current fully comparable cohorts among commercially available products. Comparisons with published data of CRS and ICANS outcomes should be interpreted with caution, as increased experience with management of these complications have led to improved outcomes (26) (29, 30). As mentioned throughout this discussion, the toxicity profile of CAR-T cell products varies with target, co-stimulatory signal and disease setting, and different preventive strategies may prove effective and safer in specific settings. Prospective research will be needed to validate the use of these preventive methods, validation of the cytokine findings will also be aided by studies in patient populations with incidence of higher-grade CRS.

The Society for Immunotherapy of Cancer (SITC) clinical practice guideline on immune effector cell – related adverse events acknowledge the reported data on pre-emptive or prophylactic use of tocilizumab, and while these strategies are not included in the recommendations, consideration of earlier tocilizumab use is recommended in elderly patients or those with extensive comorbidities (47). Our results indicate that prophylactic tocilizumab is feasible in NHL patients receiving anti-CD19 CAR-T cells with CD3ζ/4-1BB costimulatory signaling, without increased risk of ICANS and with preserved disease control. Additional studies evaluating pre-infusion CRS prevention strategies, including risk – adapted pharmacologic interventions, should be undertaken.

## Data Availability Statement

The raw data supporting the conclusions of this article will be made available by the authors, without undue reservation.

## Ethics Statement

The studies involving human participants were reviewed and approved by Institutional Review Board of University Hospitals Cleveland Medical Center. The patients/participants provided their written informed consent to participate in this study.

## Author Contributions

Wrote the paper; designed and performed research: PC, GP, FO, AS, NA, PR, and SP. Performed research: SK, JS, KZ, KB, AH, and JR. Analyzed data and designed research: MM, BD, R-PS, and ML. All authors contributed to the article and approved the submitted version.

## Conflict of Interest

BD is a previous employee of Lentigen, a Miltenyi Biotec Company, and has Patents and Royalties related to CAR-T immunotherapy.

The remaining authors declare that the research was conducted in the absence of any commercial or financial relationships that could be construed as a potential conflict of interest.

## Publisher’s Note

All claims expressed in this article are solely those of the authors and do not necessarily represent those of their affiliated organizations, or those of the publisher, the editors and the reviewers. Any product that may be evaluated in this article, or claim that may be made by its manufacturer, is not guaranteed or endorsed by the publisher.
