# Supplementary material for: Prophylactic Tocilizumab Prior to Anti-CD19 CAR-T Cell Therapy for Non-Hodgkin Lymphoma
Source: Front Immunol. 2021 Oct 12;12:745320. doi: 10.3389/fimmu.2021.745320 (PMC8546323; doi:10.3389/fimmu.2021.745320)

Supplemental Figures

Supplemental figure 1. Scatterplot of CAR-T expansion measured by qPCR, trends for generated by locally estimated scatterplot smoothing (loess), AUC of CAR-T transgene (copy/pg DNA x days) compared with Wilcoxon rank sum test, p = 0.16. AUC: Area under the curve; CRP: C reactive protein; CRS: cytokine release syndrome; LDH: Lactate dehydrogenase qPCR: quantitative polymerase chain reaction. Blue boxes, bars, dots and lines represent results of patients without CRS, red boxes, bars, dots and lines those with CRS.

Supplemental figure 1. Boxplot of time – based changes in cytokine concentrations with statistically significant differences between patients with CRS (red) and without CRS (blue). Comparisons done with Wilcoxon test. (* = $p<0.05$, ** = $p<0.01$)

Supplemental figure 1. Cytokines with statistically significant differences between patients with and without CRS (n = 13). Boxplot of cytokine concentrations at baseline, day 0, 2, 6 and 14 after CAR-T infusion in patients with CRS (red) and without CRS (blue). Comparisons done with Wilcoxon rank sum test. (* = $p<0.05$, ** = $p<0.01$). CRS: Cytokine release syndrome; INFγ: interferon gamma; IL: interleukin; MCP: monocyte chemoattractant protein; MIP: Macrophage inflammatory protein; TGFβ3: Transforming growth factor beta 3; TNFα: tumor necrosis factor alpha.

Supplemental figure 2. Cytokines with no statistically significant differences between patients with and without CRS (n = 13). Boxplot of cytokine concentrations at baseline, day 0, 2, 6 and 14 after CAR-T infusion in patients with CRS (red) and without CRS (blue). Comparisons done with Wilcoxon rank sum test. (* = $p<0.05$, ** = $p<0.01$). CRS: Cytokine release syndrome; INFγ: interferon gamma; IL: interleukin;

MCP: monocyte chemoattractant protein; MIP: Macrophage inflammatory protein; TGFβ3: Transforming growth factor beta 3; TNFα: tumor necrosis factor alpha.

Supplemental figure 3: Cytokine concentrations in patients with CRS grade ≥2 and those with grade <2 (including no CRS) (n = 13). A. Comparisons of plasma cytokine concentrations (Wilcoxon rank sum test). P values <0.05 are noted with red tiles. Not significant values (NS) are noted with black tiles. A. Median fold change from baseline (pre-lymphodepletion) over time in plasma cytokine concentrations in patients with grade ≥2 CRS (left panel) vs. those with grade 1 or no CRS (right panel). Larger increases from baseline are shown in the green – yellow spectrum. Gray tiles represent missing values.

Supplemental figure 4: Cytokine concentrations in patients with neurotoxicity vs. those without neurotoxicity (n = 13). A. Comparisons of plasma cytokine concentrations (Wilcoxon rank sum test). P values <0.05 are noted with red tiles. Not significant values (NS) are noted with black tiles. A. Median fold change from baseline (pre-lymphodepletion) over time in plasma cytokine concentrations in patients with ICANS(left panel) vs. those without ICANS (right panel). Larger increases from baseline are shown in the green – yellow spectrum. Gray tiles represent missing values.

Supplemental figure 1

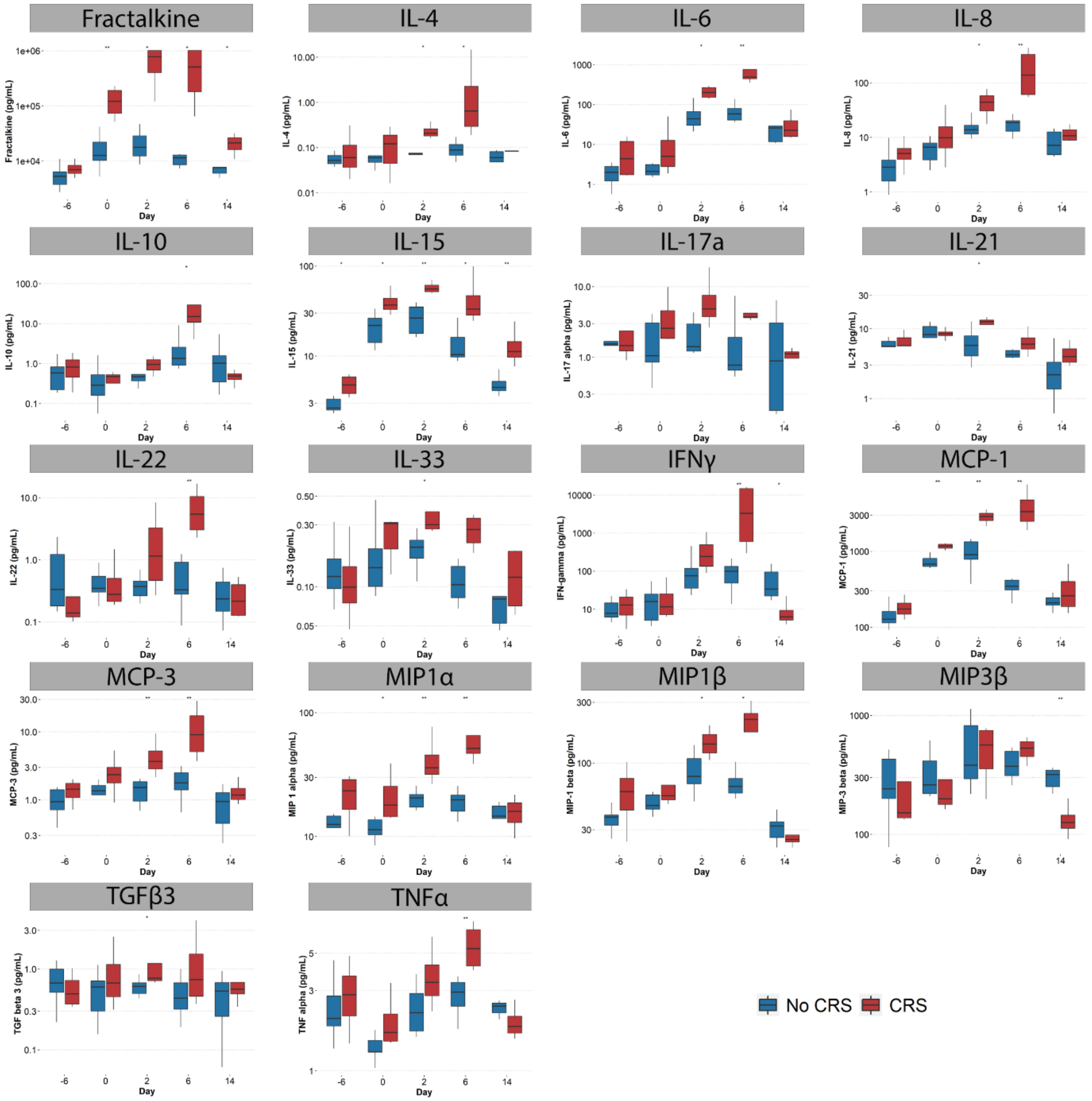

Supplemental figure 2

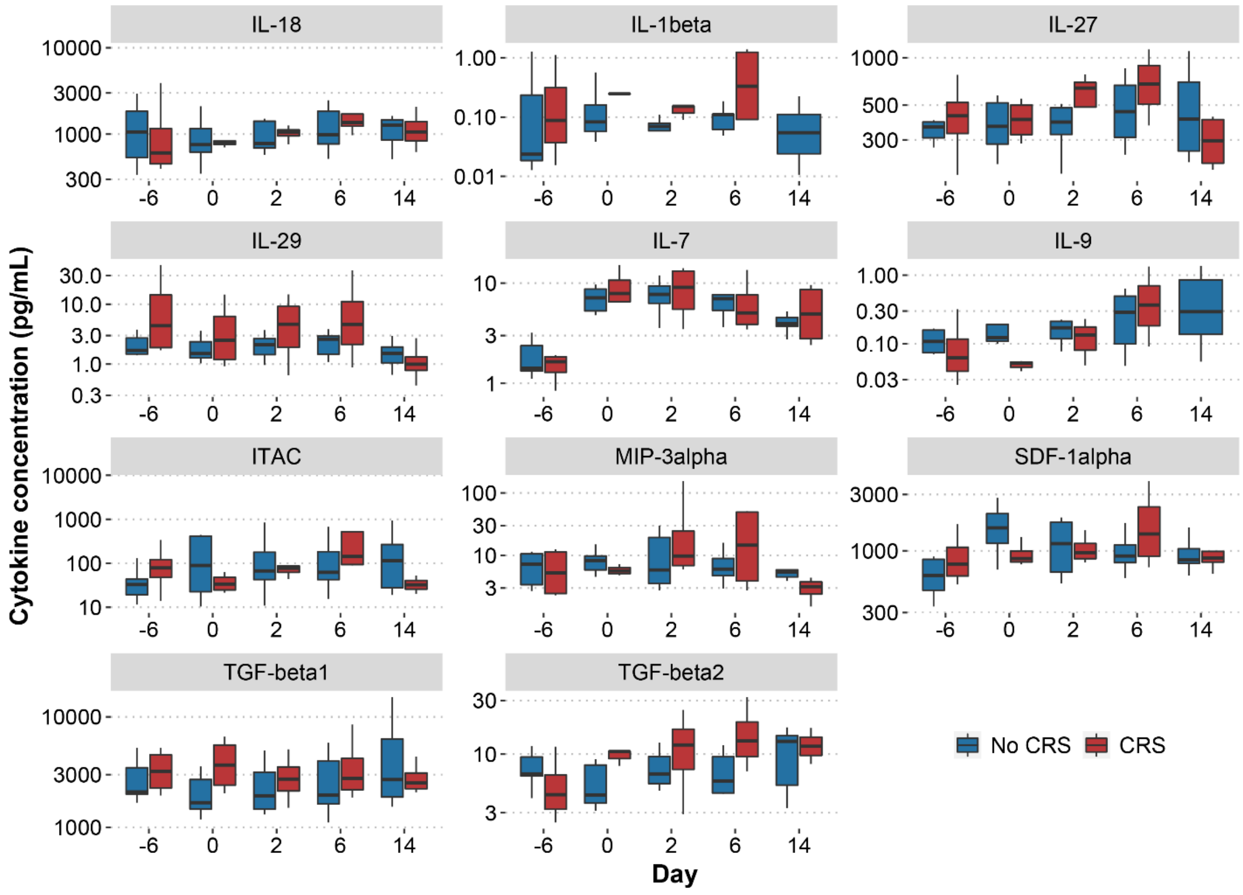

Supplemental figure 3

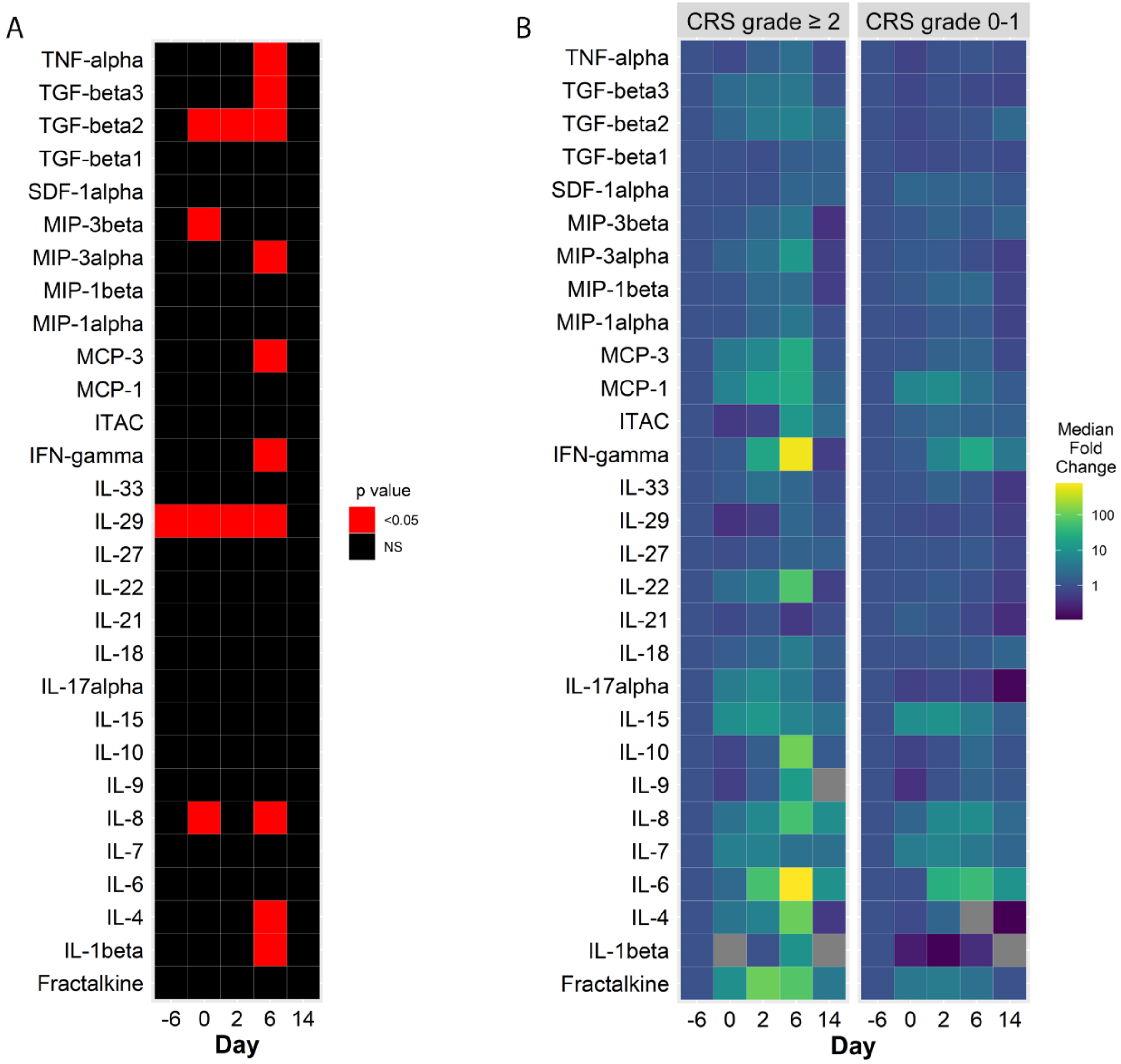

Supplemental Figure 4

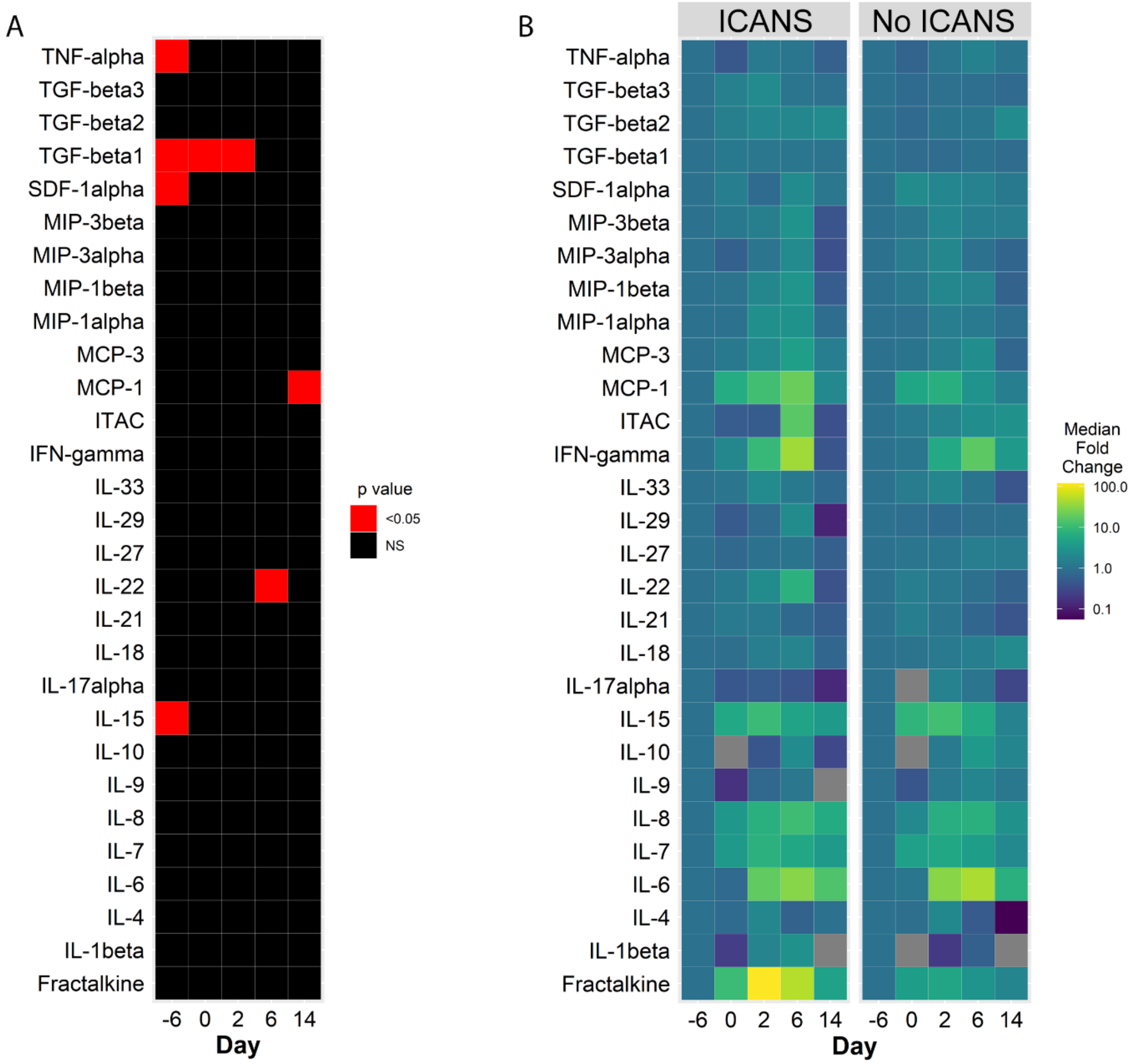

Supplement: Supplementary file 1 [file DataSheet_1.pdf]
